# Comprehensive assessment of cryogenic storage risk and quality management concerns: best practice guidelines for ART labs

**DOI:** 10.1007/s10815-018-1310-6

**Published:** 2018-09-19

**Authors:** M. C. Schiewe, M. Freeman, J. B. Whitney, M. D. VerMilyea, A. Jones, M. Aguirre, C. Leisinger, G. Adaniya, N. Synder, R. Chilton, E. J. Behnke

**Affiliations:** 1 Ovation Fertility, Newport Beach, CA USA; 2 Ovation Fertility, Nashville, TN USA; 3 Ovation Fertility, Austin, TX USA; 4 Ovation Fertility, Las Vegas, NV USA; 5 Ovation Fertility, Baton Rouge, LA USA; 6 Ovation Fertility, Indianapolis, IN USA; 7 Ovation Fertility, Los Angeles, CA USA; 8 Ovation Fertility, Cincinnati, OH USA

**Keywords:** Cryopreservation, Storage, Quality management, Embryo, Reproductive tissue

## Abstract

Recent publicized events of cryogenic storage tank failures have created nationwide concern among infertility patients and patients storing embryos and gametes for future use. To assure patient confidence, quality management (QM) plans applied by in vitro fertilization (IVF) laboratories need to include a more comprehensive focus on the cryostorage of reproductive specimens. The purpose of this review is to provide best practice guidelines for the cryogenic storage of sperm, oocytes, embryos, and other reproductive tissues (e.g., testicular and ovarian tissue, cord blood cells, and stem cells) and recommend a strategy of thorough and appropriate quality and risk management procedures aimed to alleviate or minimize the consequences from catastrophic events.

## Introduction

The cryopreservation of sperm, embryos, oocytes, and other reproductive tissues experimentally evolved in laboratory animals, domestic livestock, humans, and wildlife species as an effective tool for fertility preservation. To insure the short- and long-term maintenance of viable cryopreserved specimens, reliable cryostorage tanks capable of efficiently holding supercool liquid nitrogen (LN_2_) below −150 °C have been fabricated by creating a double metal container with an insulated, sealed vacuum air chamber between them. The traditional use of cryostorage dewar tanks of varying intermediate capacities (30–73–L), requiring manual LN_2_ level monitoring and filling, has expanded into the use of high-capacity bulk tanks (160–1000 L) with computerized monitoring and automatic filling systems [1].

For decades, animal reproduction and human fertility laboratories relied faithfully on the use of simple, dependable dewar tanks with only a rare occasion of reported tank failure. Anecdotal reports suggest most problems have arisen due to a complacency in quality control (QC) practices and a forgetfulness to refill or repair these ever-dependable vessels. If stored in obscure, low traffic locations, coupled with dependence on past reliability to maintain LN_2_ levels, these tanks are sometimes forgotten as an eternal cold chamber. Tanks can sustain extremely cold subzero internal conditions even when they seemingly exhaust the presence of LN_2_ under normal vacuum conditions, but shortly thereafter a threshold is reached with the nitrogen gas exhausted, and ambient equilibration occurs. The ability of cryogenic storage tanks to efficiently maintain LN_2_ temperatures below −150 °C, under very low liquid level conditions, is due to the contained supercooled vapor state. In turn, LN_2_ vapor (LNv) tanks have also been created to facilitate the storage of samples at risk of possible contamination, i.e., if submerged in liquid, as well as for the safe shipment of cryopreserved samples [2]. Most LNv storage tanks are reliant on auto-fill systems to maintain precious centimeters of liquid to preserve the viability of their specimens.

The introduction of high-capacity storage tank systems (i.e., bulk tanks) has increased our reliance on working parts (e.g., solenoids, valves) and digital relay systems (e.g., temperature and level sensors, alarm systems). These devices are also susceptible to equipment failure or mismanagement that can result in catastrophic specimen loss if unusual sounds and events are ignored. Although relatively rare, several tank failures have been reported [2, 3]. The recently publicized failures that caused a catastrophic loss of over 4000 patient samples in a high-capacity auto-fill tank and the near loss of embryos and oocytes in a flawed manual-fill dewar tank in another facility, have heightened the awareness of patients, laboratories, and storage entities to the potential risks and liabilities of cryostorage. For all tank systems, a series of QC steps to monitor LN_2_ levels and refill tanks is necessary for proper cryostorage maintenance. Furthermore, emergency response and back-up planning is essential to ensure appropriate measures are taken to avert or minimize the effects of potentially serious cryostorage accidents.

It was recently postulated that the frequency of moving and potentially mishandling tanks, by dropping or banging them, increases the risk of causing a damaging flaw, such as loss of vacuum pressure by puncture or compromise to welded seams and catastrophic failure [1]. This is potentially more problematic for the aluminum LN_2_ dewar and LNv dry shipper tanks. In practice, the large, steel stationary bulk tanks are a safer, more well-constructed storage system. Most reproductive laboratories, however, have found the smaller dewar tanks, typically 35–47–L, to be extremely reliable for decades and, based on space constraints, preferred to larger bulk tanks. The growing problem over cryogenic risk management is, in fact, multifaceted. How do we manage a growing population of aging tanks currently in use? Are newly manufactured tanks and monitoring devices as dependable as their predecessors? Are traditional temperature alarm systems adequate to prevent catastrophic losses? Are conventional quality control plans implemented by most IVF Labs sufficient to ensure complete protection from loss? Certainly, what is known is that all tanks will fail over time, but when? A new tank could fail to hold a full LN_2_ load for 24 h or less, while an older tank could efficiently hold its subzero temperature for decades without fault. The answer to uncertainties surrounding tank failures is the implementation of extensive, thorough, and innovative quality and risk management measures. The purpose of this paper is to comprehensively outline best practice guidelines for reliable cryogenic storage systems that minimize laboratory liability concerns and optimize patient security.

## Administration, consents, and record keeping

As the field of Assisted Reproductive Technologies (ART) has developed, cryogenic storage practices have become a subspecialty warranting independent attention and consenting. This is particularly true of large laboratories or multiple laboratories owned by corporate entities. Once included in a standard embryo and oocyte informed consent form, the risks and benefits of on-site cryostorage, or potentially at an off-site long-term cryostorage facility, now require advanced consideration. There are several reasons for this. Many established programs are (1) running out of storage space, (2) unable or perhaps unwilling to change to high-capacity bulk tanks, (3) having a significant cross-border reproductive care patient population requiring advanced arrangements and payments, or (4) motivated to share or eliminate the liability of cryostorage maintenance with a third-party contractor. In all storage consents and agreements, it is crucial to inform patients that storage devices can fail, even in lieu of comprehensive QM practices, and that they acknowledge the possibility by their signature. It is up to each program to properly execute their QM plan, for such a statement to be legally binding.

As addressed by the American Society for Reproductive Medicine (ASRM) Ethics Committee [4], informed cryostorage agreements are necessary to specifically address important issues of patient responsibilities regarding (1) embryo disposition under varying life situations (e.g., death of one or both partners), (2) agreement to maintain contact with the storage facility, (3) payment of storage fees, or (4) negotiate a new storage arrangement, such as a multi-year storage agreement. In addition, it must be clearly stated and acknowledged by the patient(s) that it is the patient’s responsibility to maintain and update current contact information, and that failure to respond to storage communications, including certified mail, may result in the activation of a release of ownership protocol and ultimately discard of their specimens. A strong stance toward the automatic discard of samples may actually hinder the desired outcome, that is, a signed agreement to either continue storage or alternatively discard specimens. If patients simply do not respond with the knowledge that the lab or storage facility “may” ultimately discard without signed consent, it is likely the long-term storage will be inefficiently extended for months, years, or perhaps indefinitely. Patients may elect to leave the decision of the disposition of their stored samples with the lab or storage facility instead of making this difficult decision themselves, particularly involving embryo cryostorage.

Record keeping in all aspects of IVF procedural practice must be thorough and complete. There must be traceability between the embryo data sheets, cryopreservation records, and cryostorage inventory. Furthermore, it is advisable that there be a duplication of records in written and/or electronic form. Additionally, verification of key points of tracking patient embryos has become an expected necessity in terms of witnessing events. In terms of specimen cryopreservation, labeling of cryodevices is a critical confirmatory step requiring standardization. Labeling must possess at least two unique identifiers, a specimen number, date, and an accurate description of the contents (e.g., 1x6AA, or HBL). Of utmost importance is the accurate description of storage location on all records, in terms of (1) tank number, (2) canister number or location (column/row/level), and (3) unique cane ID.

The maintenance of daily, weekly, and monthly QC records is a vital component of any QM system. Daily QC procedures are best performed by responsible individuals willing to strictly adhere to a standard operating procedure (SOP) and then verify entries as necessary (i.e., time, date, initials). These duties may be best rotated and delegated to different staff members for effective internal auditing purposes. The daily records, including QC forms (e.g., tank inspections and refilling), record logs (e.g., supply tank replacement), and computerized remote monitoring files (e.g., temperatures, alarms), represent documentation that is maintained on-site for a minimum of 2 years for quality assurance (QA) purposes. Alternatively, equipment records should be maintained on-site for the lifespan of the cryogenic storage tank. The actual lifespan of a cryotank may be defined by a manufacturer’s recommendation (e.g., 5 or 10 years), but is more typically defined by its functionality and useful life expectancy. Understanding that a manufacturer’s recommendation is an arbitrary conservative estimate, each program should define their own lifespan parameters. These will likely include both a maximum lifetime (e.g., 20 years), as well as a functional lifespan. The expected functionality of a tank is based on a history of repetitive QC measurements assessing external observations and a pattern of internal function (e.g., evaporation rates), as detailed below in each section.

## Cryogenic storage tank considerations

### LN_2_ and LNv phase storage tanks

High-volume LN_2_ dewars (up to 73 L) and high-capacity bulk tanks are the recommended type for long-term, indefinite storage. The more portable LN_2_ dewars are the most widely used in IVF facilities, although some have advocated the safe use of LNv tanks for sperm [5, 6] and oocytes/embryos [7–9]. In the absence of securely sealed closed-device systems, there is a theoretical risk of microbial and viral cross-contamination between specimens commonly stored in LN_2_ [7, 10, 11]. In turn, storage of samples in an LNv phase environment is undeniably safer, in this respect. However, in clinical practice, such cross-contamination of gametes or embryos has never been verified under standard cryostorage conditions [12]. Even under experimental viral challenge conditions, the transmission of disease to an offspring due to embryo cryostorage contamination has yet to manifest [13]. It may simply be that the potential viral load of a single gamete or embryo is insufficient to cause uterine or placental transfection.

The use of LNv storage is practical for the short-term storage of neat semen samples awaiting infectious disease screening test results under standard clinical conditions, as unwashed seminal fluids are a proven vector for disease transmission [7]. Use of LNv for the cryostorage of any confirmed sexual transmitted disease positive carrier samples (e.g., HIV, Hepatitis B) is also a consideration. Alternatively, the clinical use of secure, closed straw vitrification device systems (e.g., ionomeric resin, weld-sealed straws like CBS™ and Rapid-i®; [14]) represents a safe and simple approach for the cryostorage containment of high-risk samples. It does not, however, appear justified to use LNv storage to safely maintain the integrity of gametes and embryos stored in either open vitrification device systems [12] or samples with potentially defective seals (e.g., cryovials, hybrid vitrification devices; [15]). In turn, the increased cost, labor, time, and safety concerns to create and use sterile LN_2_ [16] is also therefore unnecessary. Knowing that there are documented occurrences of LN_2_ and LNv tank failures [1, 2] it is logical to conclude that the risk of catastrophic loss of vitrified samples is greater in an extremely low LN_2_ volume, LNv storage system.

A strict quality management practice of at least daily monitoring and physical tank inspections are warranted and more critical with LNv storage tanks, in contrast to liquid phase LN_2_ storage due to reduced static holding times. Therefore, one must carefully weigh the risk-benefits for LNv storage. LNv storage, based on theoretical disease transmission concerns, is not advisable since the liability of physical storage loss likely outweighs the risk analysis of sample-to-sample cross-contamination. Dry shipper LNv tanks can be used for short-term holds, as long as diligent QC practices are implemented to ensure <− 150 °C are maintained. We reiterate that the selective storage of potentially infectious samples should be quarantined in LNv and separate LN_2_ tanks, as previously mentioned above. If using liquid phase dewar tanks for quarantining samples of known infectious disease status, it is advisable to perform a periodic thorough tank cleaning/disinfection as defined by the manufacturer SOP. The justification and methodologies for disinfection of LNv/LN_2_ tanks have previously been reviewed [7, 13].

### Static holding time for cryo-storage

Cryogenic tanks are composed of both open-lid storage tanks and closed, pressurized supply tanks. Both varieties are constructed of 2–3 layers of carbon and stainless steel or aluminum, although copper- and nickel-alloys may be included. The layers are weld-sealed to create an “annular space” which is depressurized via a pump to create a high vacuum environment. The annular space is also filled with an insulating material such as perlite, silica aerogel, or fiberglass. These highly pressurized vessels are designed to minimize the transfer of heat into the inner tank cylinder to reduce vaporization, the conversion of liquid to gas when the boiling point is reached. The transition of thermal energy from the warm exterior to supercool interior is referred to as the “inleak” potential of heat transfer. Thus, the number of vacuum layers, type and amount of insulation, quality of welds, and freedom from defects (e.g., punctures), all affect the inleak potential of a tank and, for our practical purposes, the evaporation rate of LN_2_.

The ability of LN_2_ storage tanks to retain liquid volume over time (i.e., static holding time) is a critical statistic to be considered in selecting, maintaining, and retiring tanks. The manufacturer-determined static hold time varies based on the specific tank (i.e., freezer) design. The factors influencing a new tanks’ static hold time are design dimensions (height, width), lid opening width, volume capacity (L), and thermal insulation parameters. Increased insulation of the annular space and more vacuum layers improves the holding time of “high efficiency (HE)” tanks, but does so at the expense of storage capacity. In turn, laboratories must weigh the importance of monthly LN_2_ supply costs relative to long-term storage capacity needs.

Independent of the tank-type selected and used by a laboratory, it is imperative to perform operational and performance qualifications (OQ/PQ) prior to in-use application. Tank qualification begins with installation qualification (IQ). In its simplest definition, the IQ of a manual fill dewar tank involves: (1) removal from its shipping box, (2) assessment of possible damage, (3) physical inspection of the entire tank and all welds, (4) insertion of canisters and lid, and (5) placement of tank onto a roller base. Alternatively, high-capacity tanks equipped with auto-fill and alarm systems require additional detailed steps be followed in accordance with manufacturer instructions, prior to OQ.

The OQ is a validation of a new tank’s ability to retain LN_2_ volume in line with the manufacturer specified static hold time. The OQ is typically performed over a minimum 1-week duration, if urgent usage is required. Ideally, a 30-day challenge is instituted by assessing varying conditions and parameters, coupled with daily measurements assessing the maintenance of LN_2_ volume (e.g., level, weight) and temperature. For example, a combination of half-fill versus full-fill conditions can be initiated at 1-week intervals, followed by full-fill with lid off for a week and then lid on for the final week. It is important to turn off auto-fill capabilities during the experimental assessment of LN_2_ usage. An additional week or two could be added to the OQ with the auto-fill function “on,” thus validating the auto-fill feature. Testing of any alarm system and remote monitoring features, discussed below, should also be performed at this time. If tank weight measurements are implemented as a dewar tank external monitoring approach to LN_2_ evaporation rate, it is important to determine a post-fill, full LN_2_ tank weight with empty canisters and cryo-sleeve inserts only, contrasted to similar full tanks containing individual full specimen canisters to characterize minimum (empty), partial, and maximum (full) weight potentials. The latter values can be used to determine each tank’s unique evaporation rate. Once the OQ has been performed, characterized, and accepted, the tank may be approved for specimen storage.

Implementing PQ involves the daily assessment and characterization of a tank under standard working conditions. The PQ testing of a tank is based on documenting effective daily usage over the course of months. Dry shipper LNv tanks require special consideration to OQ/PQ validation following manufacturer recommended filling and pour-off procedures, as well as routine weight measurements pre- and post-shipment. In addition to standard static holding capacity determinations for validation approval, it is useful to challenge the testing conditions to assess changes in evaporation rates if the shipper tank is placed on its side or upside down for an extended period of time (i.e., 8 to 24 h). Historic changes in static hold capabilities will provide objective data to predict whether the integrity of a tank has been compromised and then formulate tank replacement decisions.

The combination of daily and weekly QC practices will be detailed below in the “Maintenance and monitoring” section. Minimum weekly LN_2_ measurement checks and/or LN_2_ filling are required. Daily temperature and/or LN_2_ measurement checks are preferable in attempting to fully characterize a tanks’ storage potential.

### Location of units

An accurate assessment of future storage needs is important in the selection and use of a cryostorage area that meets operational needs. Such needs must include an assessment of future cryostorage usage, including adequate empty back-up tank resources. Total square footage and ventilation are critical safety considerations. Ideally, a dedicated air handling system that has a complete turnover of fresh outside air, that is 100% out-take and 0% re-circulation, and is independent of the IVF lab(s) air system, is recommended. Although official standards do not exist, a minimum of 6 air exchanges per/h is recommended. For a majority of IVF programs, the above criteria are difficult to achieve in a shared building space. Alternatively, efforts should be made to effectively ventilate residual nitrogen gas out of a building, especially in an emergency low O_2_ situation.

The cryogenic storage room should be a spacious area that has observational visibility and accessibility to staff. The goal is to facilitate daily use and inspection of the tanks while not introducing security risks. It has been documented that remote storage areas, such as closets or closed rooms away from the lab, are prone to more quality management deficiencies and potential problems. In turn, the storage room should be in a well trafficked area, possessing a secure door(s) and entry limitations. Window visibility of the storage area is encouraged. Visibility provides for safe, routine surveying of the cryotanks and their environment to determine whether a possible problem condition exists, like LN_2_ leakage or a spill, before entering. The installation of a remote camera system is also recommended for continuous surveillance of the cryogenic storage area, ensuring the safety of personnel in the storage area. The location of the cryostorage room should also have easy access to LN_2_ supply delivery and removal, preferably separate from primary patient access areas.

### Emergency plan safeguards

No matter how reliable the cryotank deployed, assume that all tanks will fail at some point. For the larger bulk tank auto-fill systems, electronic components have a much shorter lifespan than a typical dewar tank. Therefore, manufacturer’s recommendations for regular calibration, repair, and part replacement (e.g., solenoids and valves) should be strictly followed. Risk management plans should include one or more adequate active empty standby tank(s) as an emergency storage back-up, of adequate capacity equal to a failed tank. The latter tank(s) must be LN_2_ equilibrated, maintained, and regularly quality controlled per SOP. In the event of an emergency, a quick and safe transfer plan should be adopted, which may temporarily involve the rapid transfer of canisters to the center of other uncompromised tanks. The emergency plan should include an adequate LN_2_ supply reserve available on-site, and/or mobile LN_2_ supply tanks available for emergency back-up.

Finally, deploy O_2_ sensor alarms in rooms where cryostorage tanks, supply tanks, and LN_2_ filling areas exist. Remote monitoring of O_2_ performs an additional safeguard to employees if an alarm notification is triggered by O_2_ levels dropping below a safe threshold, alerting individuals to potentially critical hypoxic, life-threatening conditions. All staff should evacuate premises if an actual alarm occurs and the lab director or supervisor immediately notified to survey the problem. Depending on the severity of the issue or if the problem persists, contact an emergency response unit (i.e., fire department, police) to assist with problem resolution. The availability and use of exhaust fans should be considered in an emergency response plan. A portable breathing unit may be kept outside the cryogenic storage area in case a staff member must enter to retrieve a collapsed team member from the cryogenic storage area. Safety of staff, followed by ventilation of the affected area by opening doors, windows, or activating exhaust fans, are the first priorities, followed by problem resolution until the emergency responders arrive.

## Maintenance and monitoring

### Storage tank filling

All manual-fill LN_2_ storage tanks (i.e., standard low-capacity dewar tanks; < 60–L) should be filled at least weekly using appropriate safety precautions per SOP. The safety measures routinely used include wearing insulated thermal gloves and/or liners, protective eyewear, and other personal protective equipment (PPE). Measurements of tank LN_2_ levels should be performed at least weekly, prior to all manual filling events. The purpose of these measurements is to assess the usage/evaporation rate of each tank and determine whether the liquid levels are within acceptable range limits. In addition, personnel should routinely perform spot-check assessments, possibly measurements, that the LN_2_ levels are above canister/sample device levels whenever a sample is removed from or placed into a tank.

The value of weekly measurements could be compromised under standard IVF lab cryogenic handling conditions when LN_2_ is removed from dewar storage tanks for other lab purposes, such as to fill flasks or baths for cryopreservation procedures. Alternatively, the bulk supply source (e.g., 160-L tank) can be used for these purposes, but is inefficient due to increased vapor loss and time consumption. LN_2_ removal can be recorded on a daily log and the residual proportion returned estimated; however, over time the estimated amounts would be erroneous. To minimize recording errors, including failed QC entries, it is more accurate and less laborious in terms of QC paperwork to simply use the spare emergency back-up tank as an LN_2_ reservoir. This functional use of a back-up tank facilitates a more reliable weekly assessment of primary tank evaporation rates.

Assessing the evaporation rate of a tank, in conjunction with physical inspection of welds, seals, or other visual anomalies, as detailed below, is the best gauge of a tanks’ integrity. Any tank experiencing out-of-range evaporation rates, unexplained by excessive daily usage, must be ear-marked for limited use and daily measurement monitoring performed for at least a week. If the tank remains out-of-range revealing abnormal evaporation rates, it should be retired immediately and its contents moved to the back-up reserve tank. A new tank should be purchased and OQ tested before placing into use.

In addition to a reserve storage tank, a secondary supply tank should always be maintained on-site and/or attached to a multi-port auto-fill supply manifold. Typically, IVF labs use 160- to 240-L supply tanks. Arrangements with the gas supply vendor should accommodate same-day and/or emergency deliveries, otherwise it is advisable to maintain a back-up supply tank(s) on-site. Under large cryostorage situations, if the property is suitable, the use of a large refillable LN_2_ silo supply tank (e.g., 1000 to 10,000–L) may be a safer, more practical, and cost-effective approach. Silo tanks can also be connected in series to supply LN_2_ into large cryostorage facilities. The low-pressure passage of LN_2_ from supply to storage tanks over an appreciable distance (e.g., 10′ to 100′) is best accommodated in vacuum-jacketed stainless steel piping. The latter specialized piping comes in different sizes and styles including flexible hoses. Although it is expensive, the cost of LN_2_ vapor loss to non-insulated hoses or piping must be factored into any budget.

The use of automatic filling systems has been predominately applied to high-capacity LN_2_ bulk tanks and LNv tanks. These on-off fill systems are triggered by LN_2_ level sensors and use solenoids to activate the passage of LN_2_ into the tanks through check valves. In turn, these auto-fill systems are susceptible to computer malfunction of the sensors and mechanical failure of solenoids and valves. Excessive icing and frosting of hose connections reveals problems warranting attention, as does the jack-hammering sound of a solenoid needing replacement. A routine equipment maintenance program that involves daily testing, frequent calibration, and periodic part replacement is critically important to stay ahead of unexpected failures. Test the auto-fill supply daily by opening the insulated lid and confirming an auto-fill response. Additionally, the manual override should be activated to conduct a fill test. As mentioned above, auto-fill tanks require a back-up LN_2_ supply at all times.

### Manual tank monitoring and physical assessments

As we discussed above, manual measurements of LN_2_ levels should be performed daily upon opening a tank in use or at least weekly prior to filling it. In addition, each tank and the component parts of the fill system must be visually inspected and physically touched daily to thoroughly inspect each tank. Bulk tanks encased by a decorative case are visually pleasing but less functional in terms of preventive maintenance practices. The physical inspection of a tank should involve a multifaceted inspection approach that follows the “LIFES” acronym. LIFES stands for the detection of the following:

1. Leakage—LN_2_ escaping from hoses or a tank warrants immediate attention and resolution;
2. Ice—its presence on a tank, hose connection, valves, or electronic connection should never be ignored, and efforts should be made to determine the root cause of “icing”;
3. Frost—its unusual presence on tank, hoses, and lids (i.e., all connections) warrants investigation;
4. Evaporation and other suspicious conditions considered odd or unusual, like condensation, should be recorded and further observed for possible resolution if the problem persists. Condensation can be detected as “sweating” outer surfaces of a tank and/or water pooling on the floor, which may indicate loss of tank vacuum pressure; and
5. Sounds—any odd/unusual sound (e.g., hissing, jack-hammering) should be identified and resolved.

Note that the neck region of aluminum LN_2_ dewar and LNv shipper tanks are particularly vulnerable to cracks in the welds that could compromise the vacuum. Therefore, close inspection of condensation, outer welds, and inner neck integrity, as possible, is vital to daily tank QC.

Any occurrence of the above possible events would then mandate the implementation of a “Corrective Action-Preventive Action” (CAPA) plan. The immediacy of the response is dependent on the severity of the impending problem. Overall, the daily physical inspection of storage tanks should focus on welds, especially around the neck, lid attachment, hoses, display panel, and all other connections. As part of the QM program, it is critically important to create a CAPA report to proactively address any potential problem(s) as part of standard QC practice. The previously discussed visual LIFES cues should never be ignored. These observations coupled with LN_2_ level measurement trends and/or other remote monitoring measurements will provide invaluable information to help determine when to retire a storage tank and move all samples to a new qualified tank, and when to replace dry LNv tanks from routine shipment use.

### Remote monitoring systems

The most common form of monitoring system for LN_2_ storage tanks is temperature, both for dewar tanks and high-capacity tanks. Continuous monitoring of temperature is performed in association with a remote alarm system activated by out-of-range measurements. Consistency of temperature sustainability below a threshold set-point (e.g., −185 °C, −180 °C, −175 °C) is an excellent QC/QA measure which documents tank stability; however, temperature measurements by themselves are a poor gauge of LN_2_ levels, except at critically low levels requiring immediate action (i.e., LN_2_ filling). Surprisingly low levels of LN_2_, in a tank with an intact vacuum, can maintain a supercool vapor environment which safely preserves samples; however, in very short order, perhaps < 24 h, safe temperature below -150 °C can rise to ambient temperatures once dry. The latter condition occurs much more rapidly in a tank whose vacuum has been compromised, which is accelerated in smaller capacity dewar tanks. Low level LNv tanks are particularly susceptible to catastrophic failure if a critical level of LN_2_ is not maintained by its auto-fill system, as previously mentioned. Therefore, temperature monitoring alone is not considered a particularly effective gauge to avert a crisis.

To optimize the sensitivity of the temperature monitoring system, it is important to secure the probe high in the tank and a low sub-zero temperature set as the threshold (e.g., −185 °C). Although the latter setting will potentially increase your emergency response time, it will likely also increase the occurrence of false alarm responses, e.g., if a lid is removed too long and ambient air settles in over the vapor phase. Daily control temperature measurements should be recorded, and calibrations routinely performed when out-of-range greater than 2 consecutive days. Furthermore, routinely test the alarm systems, as discussed below.

A common alternative or secondary monitoring system should sense LN_2_ levels. In dewar tanks, these sensors are typically a single central probe through the lid to alert users when liquid levels go below a predetermined level (e.g., top of canisters), indicating a need to refill. In bulk tanks, level sensors (e.g., float switches, ultrasonic device) are commonly used to activate and de-activate the auto-fill system to maintain a consistent LN_2_ level. Some level sensors (e.g., capacitive and ultrasonic) are now used to remotely measure changes in LN_2_ levels to evaluate tank evaporation rates.

The use of weight to determine LN_2_ fill capacity has been a standard QM practice for LNv dry shipper tanks, typically by commercial third party cryogenic companies. Comparing pre-ship and post-return weights are a routinely applied QC practice that provides valuable secondary information to the maintenance of sub-zero temperature logging for PQ determinations. Static LN_2_ holding capacity (max days) should be correlated to daily weight measurements and internal chamber temperature measurements. Applying the concept of weight measurements, or perhaps pressure sensors, to dewar tanks (20–73–L) could represent a practical, safe, and accurate way to externally monitor evaporation rates. Through properly conducted OQ testing (i.e., accounting for empty to full capacity canisters), it is possible to correlate fluid levels to changes in tank weight or pressure. In combination with a remote monitoring system, real-time evaporation rate determinations could be an invaluable resource for (1) anticipating tank failures and objectively justifying tank retirements and (2) sensing problems and optimizing response-time intervals under emergent conditions. This type of system would require semi-annual calibration of the weight scale(s). The scale itself could rest between the roller base and the tank or be adapted as its own independent roller base. This innovative system is currently under development, with the intent to remotely network to a continuous, cloud-based data recording/alarm system.

An additional source of valuable information is real-time and time-lapsed video documentation. The use of multiple wireless cameras around the cryogenic storage area creates a vital source of continuous data that can be viewed actively as well as used as a library resource. Real-time viewing can allow an individual to periodically audit daily activities, e.g., visualize digital control panels, and staff performing QC procedures, and perhaps more importantly to safely survey the area in the event of an emergency O_2_ alarm and/or LN_2_ spillage situation. Alternatively, reviewing saved video footage provides for precise determination and documentation when possible incidents occur. Therefore, video monitoring is a critical component to facilitate root cause assessments (RCA) in an optimized total quality management (TQM) plan [17].

### Remote alarm systems

A telephone-based alarm call-out system (i.e., Sensaphone) has been effectively used in the IVF industry for decades in the monitoring of incubator function and to alert staff of low-level LN_2_ conditions. This is a wire-driven monitor/alarm system with physical space and phone line limitations. Typically, it has 4 “alert zones” that alarm sensors hard-wired to lab equipment. When an alarm activates the sensor attached to an alert zone, the specific piece of equipment causing the alarm is identified by the alert zone it is connected to. If more than four pieces of equipment need monitoring, multiple sensors can be connected to each alert zone. The group of equipment causing the alarm would be identified, instead of an individual piece of equipment, by the alert zone they were connected to. The latter daisy chain of sensors potentially slows emergency response time as the actual tank in distress still needs to be identified onsite.

Newer wireless monitoring/alarm-based units offer a more versatile solution with the ability to gather and store large amounts of data while delineating normal from abnormal conditions among individual tanks. In the event of an alarm, the Sensaphone, or other web-based information gathering QC applications, can automatically call preprogrammed telephone numbers in a directed order, e.g., IVF lab phone number, on-call phone number, and lab personnel phone numbers. These alarm notification systems, as well as the newer Wi-Fi and e-cloud texting and email-linked systems, will continue to call and send messages in a set order until someone cancels the alarm either directly in the lab, or by communicating by phone or computer directly with the alarm system. The Sensaphone is a hard-wired system that has battery back-up, thus allowing it to function if the building loses power.

Although the wireless monitoring systems can also function during black-out conditions, their reliance on batteries makes them vulnerable to the strict adherence to a QM policy of battery replacement. Furthermore, wireless alarm systems are vulnerable to Internet disruptions. It is important to have a reliable, dedicated Wi-Fi connection or ensure notice is provided when IT personnel service shared computer systems potentially triggering false alarms/disabling the system temporarily. Test all alarm systems, particularly O_2_ monitoring of personnel safety, regularly (e.g., daily to weekly) as standard QC practice to ensure proper functioning. Additionally, the call-out system should be tested regularly (i.e., weekly, monthly) to confirm their effectiveness and personnel responsiveness. Such testing can help identify software issues and equipment faults. Also, routinely clear alarm logs to ensure that sufficient storage exists, whereas exceeding maximum storage space could stop current event recording.

### Alarm response

Once the tanks alarms are connected and SOP is being followed for monitoring, filling, and regular alarm testing, laboratories are prepared to handle an emergency situation. If this occurs during the middle of the day, when IVF staff are present, the situation can be handled expediently: the source of the alarm can be located and, if the tank in question is determined to be in failure, the frozen specimens can be moved to one of the fully-charged backup tanks. The failed tank is removed from service and an order is placed for a replacement tank. However, if the alarm is triggered at a time when IVF staff are not on-site, the situation becomes more critical. The alarm activates the automatic preprogrammed call system and, ideally, the responsible staff member on-call will respond to the alarm within 15 to 60 min, depending on how far they live from the laboratory. The situation is then handled similarly to the response to a daytime alarm. What if the embryologist on-call lives an hour away? By the time he or she reaches the lab, the frozen samples could be compromised. This is something that needs to be well thought out in advance of an actual emergency situation and included in the SOP. Often, call lists are prioritized by ranking individuals whom live closest to the laboratory. If some of the embryologists live a great distance from the lab, a plan should be developed to decrease their response time to the lab in case such an event occurs. For instance, the on-call embryologist could contact another embryologist or designated, trained individual who could respond more rapidly before their arrival.

Identifying the tank causing the alarm is imperative to optimizing response time. As mentioned earlier, multiple sensors connected to one “alert zone” on an older alarm monitoring system is potentially problematic. In the latter situation, each piece of equipment should be fitted with its own alarm that sounds and lights up for quick identification in an alarm situation.

### Sample management and inventory

As part of standard cryopreservation procedures for the labeling and accessioning of gametes, embryos, and reproductive tissues, it is imperative to verify and witness all sample-labels prior to storage. As mentioned earlier, labels should possess two unique identifiers (e.g., patient name, cycle ID number), date of cryopreservation, and sample description (e.g., embryo number, stage, quantity if > 1). In the accessioning of samples, the information should be accurately recorded by at least two separate methods, e.g., written forms, log books, and electronic files input. The storage location must be identified and recorded, indicating the sample ID (i.e., cane, visotube, or box) and its location (i.e., tank number, canister number, level).

A reliable inventory is based on an accurate recording of cryostorage location for each sample cryopreserved. Regular thawing events performed in an IVF laboratory provides an excellent source of spot check verifications when organized as a QC/QA process. Furthermore, confirmation of sample locations relative to the routine process of discarding patient consented samples is another valuable spot check inventory resource that should be integrated into a QM plan. In addition to maintaining a daily QC log of sample disposition relative to their accuracy of location, random sampling (ID verification) of a given number of samples on a regular basis (monthly, quarterly) is a reassuring QC step in a comprehensive QM plan. The latter verification procedure is less desirable to thawing /discarding procedures, as it may unnecessarily expose viable samples to brief but possible changes in temperature. Perform all verifications in tandem with a witnessing staff member. Any sample confirmed to have been misidentified or incorrectly located warrants a CAPA report. The latter situation, especially if replicated, may mandate the implementation of a complete inventory check.

Complete inventory verifications can be extremely time consuming and is one of the most labor-intensive laboratory QC processes. The complete process requires a preliminary organization of records to be verified by tank, and a QC measure implemented by at least two staff members participating in a “call out” confirmation QA process of sample ID, location and number of straws, vials, or cryo-devices present. An effective way of managing such a task is the performance of a “rolling inventory” of tanks. This involves surveying the entire contents of one or more tanks on a monthly basis, as to not overwhelm staff time over a block of time. Rolling inventories on a monthly or quarterly basis are an alternative to a labor intensive annual inventory. Evaluation of the risk-benefits of performing complete or partial inventories must be seriously assessed, especially in regard to open vitrification devices. An efficient “spot check” inventory QM practice, assuring 100% accuracy on a monthly basis, is a far more cost-effective approach to verify the cryo-inventory overtime.

## Other system management practices and control considerations

In essence, the general QM plans instituted by all IVF laboratories must be equally comprehensive regarding cryostorage risk management and more so. As we have all been reminded of in this past year, the failure of cryogenic equipment, can have catastrophic outcomes to a patients’ and business’ well-being. Strict preventive maintenance of equipment and comprehensive SOP’s, as alluded to in this paper, are vital to the TQM of cryogenic storage. Boredom and staff complacency in the implementation of mundane daily QC steps can eventually result in serious repercussions. Therefore, cross-checking staff performance of QC events, as well as periodic audits, both external and internal, should be performed as part of the TQM plan. This will no doubt demand more time and labor, including weekends, to ART laboratory staff which in many cases is already stretched thin due to the increasing procedural demands of blastocyst biopsy and vitrification-all cycles. Physicians and executive officers must be willing to support staff resources to optimize implementation of the QM plan.

As mentioned earlier, and as recent events have proven, all tanks will fail at some point. As one survey has shown, up to 11% of industry participants experienced some form of tank failure [3]. A more recent webinar viewed by over 100 participants revealed that 24 of 63 (38%), 11 of 67 (16%), and 5 of 61 (8%) of the respondents experienced a failed LNv dry shipper, manual fill dewar tank, or auto-fill bulk tank, respectively. Overall, 33 of 65 (51%) pollsters experienced a tank failure, of which 15 of 33 (45%; 23% overall) suffered patient sample loss [18]. Although the webinar poll may represent a biased subsampling of pollsters, it confirms that LN_2_ tanks can and will fail. Considering that 55% of the “failed tanks” occurred without significant specimen loss, it further verifies the importance of emergency plans that include a SOP and performing practice drills. In conjunction with staff preparedness, employee safety and training are an integral part of handling emergency situations that could involve life-threatening hypoxic conditions. To optimize the protection of human resources and patients’ cryopreserved materials, system redundancy planning is essential. Finally, in response to alarms and unusual conditions, an organizational hierarchy of responsible individuals must be established, understood, and tested periodically.

## Summary

In light of recently publicized storage failures, inspections conducted by regulatory organizations (FDA, State Tissue Banking, CLIA) and laboratory accrediting bodies (AATB, CAP, JCAHO, TJC) will no doubt display a heightened interest in a facilities cryostorage QM plan. With an emphasis on risk assessment of tank failures, remote monitoring and alarm systems will become necessary in the continuous assessment of tank functionality. Failures can occur when alarms are “turned-off” and a critical issue is ignored. It is more likely that alarm systems and tanks do not fail, but complacency can result in failure. Therefore, it is essential to act and resolve repetitive signals of an impending problem by implementing a CAPA plan.

Although not an absolute regulatory requirement, if strict daily QC/QA tank management practices are adhered to, they do offer irreplaceable documentation in the event a tank failure. The daily physical inspection of tanks can provide insights into symptoms of possible problems with vacuum seals or faulty equipment. Any tank confirmed to have a potential problem must undergo re-validation testing, and have its samples potentially transferred to a back-up unit. If a comprehensive QM plan is established but not routinely followed and strictly applied, then the laboratory may have increased liability if a tank fails. The human element is the greatest risk to a biorepository’s QM system, requiring the application of secondary and tertiary QC measures.

Over the past two decades, embryologists have set out to improve QC practices and technologies in the IVF lab to enhance embryo development, selection, preservation, and implantation. Meanwhile, many have been maintaining the status quo regarding their cryostorage management. With aging cryogenic storage equipment and a growing frozen inventory, the time has come for the IVF industry to prioritize cryogenic risk assessments. We can learn from the TQM measures applied by commercial cryobanks and begin to innovate how we anticipate and respond to impending failure to prevent catastrophic losses and liability problems within the ART laboratory. Recent events have given the industry a wake-up call to seriously think about and actively improve cryogenic QM practices.
